# Involvement of Macrophages in the Pathogenesis of Familial Amyloid Polyneuropathy and Efficacy of Human iPS Cell-Derived Macrophages in Its Treatment

**DOI:** 10.1371/journal.pone.0163944

**Published:** 2016-10-03

**Authors:** Genki Suenaga, Tokunori Ikeda, Yoshihiro Komohara, Koutaro Takamatsu, Tatsuyuki Kakuma, Masayoshi Tasaki, Yohei Misumi, Mitsuharu Ueda, Takaaki Ito, Satoru Senju, Yukio Ando

**Affiliations:** 1 Department of Neurology, Kumamoto University Graduate School of Medical Sciences, Kumamoto, Japan; 2 Department of Clinical Research Support Center, Kumamoto University Graduate School of Medical Sciences, Kumamoto, Japan; 3 Department of Cell Pathology, Graduate School of Medical Sciences, Kumamoto University, Kumamoto, Japan; 4 Department of Biostatistics Center, Kurume University, School of Medicine, Kurume, Japan; 5 Department of Pathology and Experimental Medicine, Kumamoto University Graduate School of Medical Sciences, Kumamoto, Japan; 6 Department of Immunogenetics, Kumamoto University Graduate School of Medical Sciences, Kumamoto, Japan; Universite Claude Bernard Lyon 1, FRANCE

## Abstract

We hypothesized that tissue-resident macrophages in familial amyloid polyneuropathy (FAP) patients will exhibit qualitative or quantitative abnormalities, that may accelerate transthyretin (TTR)-derived amyloid deposition. To evaluate this, we examined the number and subset of tissue-resident macrophages in heart tissue from amyloid-deposited FAP and control patients. In both FAP and control patients, tissue-resident macrophages in heart tissue were all Iba^+^/CD163^+^/CD206^+^ macrophages. However, the number of macrophages was significantly decreased in FAP patients compared with control patients. Furthermore, the proportion of intracellular TTR in CD14^+^ monocytes was reduced in peripheral blood compared with healthy donors. Based on these results, we next examined degradation and endocytosis of TTR in human induced pluripotent stem (iPS) cell-derived myeloid lineage cells (MLs), which function like macrophages. iPS-MLs express CD163 and CD206, and belong to the inhibitory macrophage category. In addition, iPS-MLs degrade both native and aggregated TTR in a cell-dependent manner *in vitro*. Further, iPS-MLs endocytose aggregated, and especially polymerized, TTR. These results suggest that decreased tissue-localized macrophages disrupt clearance of TTR-derived amyloid deposits, leading to progression of a pathological condition in FAP patients. To improve this situation, clinical application of pluripotent stem cell-derived MLs may be useful as an approach for FAP therapy.

## Introduction

Familial amyloid polyneuropathy (FAP) is a neurodegenerative disease caused by deposition of mutated transthyretin (TTR)-derived amyloid fibrils in several organs [1]. To date, there are already more than 130 reported mutations [1, 2]. Amyloidogenic TTR (ATTR) V30M was first identified, and is the most common mutation worldwide [3–5]. In the early stage, FAP ATTR V30M patients present with polyneuropathy, thermal hyperalgesia, dysesthesia, gastrointestinal symptoms, orthostatic hypotension, and numbness in the hands and feet [6]. With disease progression, they exhibit visual impairment, heart and chronic renal failure, and death usually results within 10 years [6].

TTR is a plasma protein synthesized mainly in the liver, choroid plexus of the brain, retinal pigment epithelial cells, and pancreatic α-cells [7–11]. Normally, TTR is present as a tetramer in blood, and transports thyroxine and retinal binding protein/vitamin A [12, 13]. FAP patients and carriers exhibit a mixture of wild-type and mutated TTR; therefore, tetrameric complexes composed of a combination of wild-type and mutant TTR are found in the body [14]. It has been suggested that heterotetrameric TTR is unstable and easily dissociates into monomeric TTR [15], which likely induces conformational changes [15]. Based on this theory, tafamidis and diflunisal have recently been used as stabilizers of circulating tetrameric TTR [6]. These drugs inhibit conformational changes and fibril formation of TTR by preventing dissociation from heterotetrameric TTR into monomeric wild-type and mutated TTR [16]. Although these drugs are useful for the prevention of deposited TTR in tissue, the elimination effect of already deposited TTR-derived amyloid fibrils is unknown.

Alternatively, for the purpose of removing mutant TTR from blood, liver transplantation (LT) has been performed as a FAP treatment, and shown success in prolonging survival of FAP ATTR V30M patients [17, 18]. Importantly, the loss of already deposited TTR-derived amyloid fibrils was observed in several organs after LT [19, 20]. This suggests that, if supply of mutated TTR declines, FAP patients are innately capable of treating mutated-derived TTR amyloid fibrils that are already present. However, a recent study found that LT therapy did not prevent progression of cardiac amyloidosis in FAP ATTR V30M patients [21, 22]. In addition, we have previously shown that FAP ATTR V30M patients shift to systemic wild-type-derived TTR amyloid formation after LT [19, 21]. Altogether, these results suggest that TTR removal ability is weak in FAP patients, irrespective of LT.

Macrophages are innate immune cells that are widely distributed in various tissues [23]. These cells exhibit phagocytotic function against invading and dying cells, and regulate tissue homeostasis [23]. Macrophages are classically divided into two groups. Inflammatory macrophages (classical macrophages) are induced by pathogen-associated molecular patterns such as lipopolysaccharide and interferon-γ [24]. These macrophages release inflammatory products such as interleukin (IL)-12, nitric oxide, and reactive oxygen species, resulting in increased bactericidal activity [25]. Conversely, inhibitory macrophages (alternative macrophages) are induced by macrophage colony-stimulating factor (M-CSF), IL-4, IL-10, and IL-13. Inhibitory macrophages release anti-inflammatory cytokines such as IL-10 and transforming growth factor-β [24, 26]. CD163 and CD206 are representative cell surface markers on inhibitory macrophages [27–29].

We recently established *in vitro* methods to generate myeloid lineages (MLs) from induced pluripotent stem (iPS) cells, which are characterized by pluripotency and an infinite propagation capacity [30]. We found that iPS cell-derived myeloid cells (iPS-MLs) are phagocytic, and exert therapeutic effects in a mouse model of Alzheimer’s disease by degrading β-amyloid [31, 32].

As well as Alzheimer’s disease, iPS-MLs may also act as therapeutic agents for deposited TTR-derived amyloid fibrils, and thereby alleviate FAP pathology. Therefore, in the present study, we examined the phenotype of tissue-resident macrophages in heart tissue from FAP patients and controls. We found that tissue-resident macrophages are CD163 or CD206 positive, with a lower number in FAP patients compared with control patients. In addition, the frequency of TTR uptake in CD14^+^ monocytes derived from peripheral blood mononuclear cells (PBMC) was decreased in FAP patients compared with healthy donors (HD). Furthermore, we found that iPS-MLs degrade native and aggregated TTR, and endocytose aggregated TTR *in vitro*. These results suggest that tissue-resident CD163^+^ or CD206^+^ macrophages are involved in FAP pathogenesis, and iPS-ML therapy may be a novel and rational approach for the treatment of FAP.

## Materials and Methods

### Patients and blood samples

Autopsy specimens were evaluated from 16 non-LT FAP ATTR V30M patients and 11 control patients without heart disease, who were autopsied at Kumamoto University Hospital between 1987 and 2012. The specimens were prepared from formalin-fixed, paraffin-embedded tissue blocks and subsequently stored. Clinical information on these patients is shown in Table 1. For flow-cytometry analysis, PBMC were separated from blood of 15 non-LT ATTR FAP patients (13 V30M, one Y114C, and one I107V) and 15 HD. For TTR staining experiments, CD14^+^ cells were purified from PBMC of six non-LT ATTR V30M FAP patients and seven HD. These samples were collected at Kumamoto University Hospital between 2012 and 2016. Written informed consent was obtained from HD and patients for participation in this study, and all experiments using human samples were conducted in accordance with The Declaration of Helsinki and the approval of the Institutional Review Board of Kumamoto University (Permit Number: 1087).

**Table 1:** Clinical Characteristics of FAP ATTR V30M and Control Patients.

Patient	Sex	Age at FAP onset	Age at death	Time from FAP onset to death	Heart weight (g) at autopsy	Main disease
**FAP 1**	M	54	61	7	690	FAP
**FAP 2**	F	28	38	10	240	FAP
**FAP 3**	M	60	67	7	438	FAP
**FAP 4**	M	29	38	9	420	FAP
**FAP 5**	M	30	36	6	380	FAP
**FAP 6**	F	55	63	7	460	FAP
**FAP 7**	M	35	51	14	400	FAP
**FAP 8**	F	63	69	5	240	FAP
**FAP 9**	F	52	60	7	395	FAP
**FAP 10**	F	34	47	13	450	FAP
**FAP 11**	M	23	38	15	390	FAP
**FAP 12**	M	31	42	11	340	FAP
**FAP 13**	M	23	34	11	490	FAP
**FAP 14**	F	28	42	14	320	FAP
**FAP 15**	M	25	38	13	330	FAP
**FAP 16**	F	37	47	10	300	FAP
**Control 1**	F	NA	58	NA	430	Gastric cancer
**Control 2**	M	NA	59	NA	320	Colon cancer
**Control 3**	M	NA	54	NA	320	Intrahepatic cholangiocarcinoma
**Control 4**	M	NA	43	NA	300	Hepatocellular carcinoma
**Control 5**	F	NA	52	NA	ND	Lung cancer
**Control 6**	M	NA	54	NA	500	Lung cancer
**Control 7**	F	NA	46	NA	180	Gastric cancer
**Control 8**	M	NA	56	NA	254	Esophageal cancer
**Control 9**	M	NA	53	NA	294	Multiple myeloma
**Control 10**	M	NA	57	NA	230	Malignant lymphoma
**Control 11**	M	NA	59	NA	190	Lung cancer

M, male; F, female; NA, not applicable; ND, no data.

### Generation of aggregated TTR

Recombinant human wild-type and V30M (mutated) TTR were purchased from Wako (Osaka, Japan). To generate aggregated wild-type or mutated TTR, each TTR was incubated at pH 4.0 for 24 h [33].

### Congo red and immunohistochemistry staining

Formalin-fixed heart and kidney samples were serially sectioned at a thickness of 4 μm and adhered to microscope slides. Slides were stained with haematoxylin-eosin (HE) and alkaline Congo red. For immunohistochemistry staining, tissue samples were deparaffinized and treated with methanol containing 0.3% H_2_O_2_ for 30 min to inactivate endogenous peroxidase [34]. Normal goat serum (Dako, Glostrup, Denmark) or Blocking-One (Nacalai Tesque, Kyoto, Japan) was used to block nonspecific background staining. Monoclonal mouse anti-human CD68 antibody (Clone: G-M1; Dako), monoclonal mouse anti-human CD163 antibody (Clone: 10D6; Novocastra, Newcastle upon Tyne, UK), monoclonal mouse anti-human CD206 antibody (Clone: 5C11; Acris Antibodies, Herford, Germany), polyclonal rabbit anti-human Iba1 antibody (Wako), and polyclonal goat anti-human CD3-ε antibody (Clone: M-20; Santa Cruz Biotechnology, CA, USA) were used as primary antibodies. The secondary antibodies were polyclonal goat anti-rabbit (Dako), polyclonal goat anti-mouse (Dako), and polyclonal rabbit anti-goat antibody conjugated with horseradish peroxidase (HRP) (Dako). Reactions were visualized using the DAB Liquid System (Dako). Five visual fields from each stained section were randomly chosen and the number of each positive cell type counted by two observers. The average count number per stained section was calculated. For double-immunohistochemical staining, a mixture of anti-human Iba1 antibody and either anti-human CD163 or CD206 antibody was used as the primary antibody solution. Simple Stain Mouse MAX-PO (Nichirei, Tokyo, Japan) was reacted and visualized by DAB, and then Simple Stain RAT MAX-PO was reacted and visualized by Fast Blue BB (Sigma-Aldrich, St Louis, MO, USA).

### Cell culture

SH-SY5Y cells, a human neuroblastoma cell line (ATCC, Manassas, VA, USA), were cultured in DMEM medium (Gibco, Carlsbad, CA, USA) supplemented with 10% FBS (Sigma-Aldrich) and 100 μg/mL of penicillin-streptomycin (Gibco) at 37°C in 5% CO_2._ iPS-MLs were maintained as described previously [30]. Briefly, iPS-MLs were cultured in MEMα medium (Wako) supplemented with 10% FBS, 100 μg/mL of penicillin-streptomycin, 25 ng/mL of human M-CSF (Prospec-Tany Technogene, Rehovot, Israel), and 50 ng/mL of human granulocyte M-CSF (Prospec-Tany Technogene) at 37°C in 5% CO_2._ To inhibit proliferation of cultured iPS-MLs, mytomycin C (Sigma-Aldrich) pre-treated iPS-MLs (1 × 10^4^, 5 × 10^4^, or 1× 10^5^ cells/well) or SH-SY5Y cells were cultured with 800 nM native (wild-type or mutated) or aggregated TTR in 96-well flat-bottomed plates in FBS-free conditions. Three days later, culture supernatants were analyzed by enzyme-linked immunosorbent assay (ELISA) and western blotting, and cultured cells used for immunohistochemistry.

### Separation of CD14^+^ monocytes from peripheral mononuclear blood cells

PBMC were isolated from human blood using Ficoll-Paque (GE Healthcare, Buckinghamshire, UK). PBMC were incubated with FcR blocking regent (Miltenyi Biotec, Bergish Gladbach, Germany) on ice for 10 min, and then CD14^+^ monocytes purified using the MACS cell sorting system (Miltenyi Biotec).

### Flow-cytometry analysis

PBMC or iPS-MLs were incubated with FcR blocking antibodies (eBioscience, San Diego, CA, USA) on ice for 15 min, and subsequently labeled with specific antibodies at 4°C. The following antibodies were used: phycoerythrin (PE)-conjugated anti-human CD11b (Clone: ICRF44; eBioscience), fluoresceinisothiocyanate (FITC)-conjugated anti-human CD45 (Clone: HI30; BD Biosciences, Bedford, MA, USA), PE-conjugated anti-human CD33 (Clone: WM53; BD Biosciences), FITC-conjugated anti-human CD14 (Clone: 61D3; eBioscience), PE-conjugated anti-human CD16 (Clone: eBioCB16; eBioscience), PE-conjugated anti-human CD163 (Clone: RM3/1; BioLegend, San Diego, CA, USA), and PerCP-conjugated anti-human CD206 (Clone: 15–2; BioLegend). FITC-, PE-, and PerCP/Cy5.5-conjugted mouse IgG1 κ (Clones: P3.6.2.8.1 and MOCP-21) were used as isotype-matched controls. Stained cells were analyzed using FACS Calibur (BD Biosciences).

### Enzyme-linked immunosorbent assay

After culturing iPS-MLs or SH-SY5Y cells with the appropriate TTRs for 3 days, TTR concentration in each culture supernatant was measured by ELISA. Briefly, 96-well plates were coated overnight at 4°C with supernatants collected in carbonate buffer. The next day, plates were blocked with coating buffer containing 0.5% gelatin for 1 h at room temperature. To detect TTR, polyclonal rabbit anti-human TTR antibody (Dako; diluted 1:1000) was used as the primary antibody, and polyclonal goat anti-rabbit IgG antibody conjugated with HRP (Dako; diluted 1:5000) as the secondary antibody, which were sequentially incubated for 1 h at room temperature. After incubation with SureBlue™ TMB Microwell Peroxidase Substrate (KPL, Gaithersburg, MD, USA), absorbance was detected at 450 nm.

### Western blotting analysis

After iPS-MLs or SH-SY5Y cells were cultured with the appropriate TTRs for 3 days, culture supernatants were mixed with sample buffer (Bio-Rad Laboratories, Hercules, CA, USA) containing 2-mercoptoetanol, and boiled at 95°C for 5 min. Resultant samples were separated by SDS-PAGE and transferred to PVDF membranes (Bio-Rad). Polyclonal rabbit anti-human TTR antibody (Dako; diluted 1:1000) was used as the primary antibody, and polyclonal goat anti-rabbit antibody conjugated with HRP (Dako; diluted 1:5000) as the secondary antibody. The reaction was visualized using ECL prime reagents (GE Healthcare, WI, USA), and detected using a LAS-4000EPUVmini (GE Healthcare).

### TTR staining in CD14^+^ monocytes and iPS-MLs

To stain CD14^+^ monocytes, iPS-MLs and SH-SY5Y cells were cultured with the above-described TTRs, and re-suspended at a density of 2 × 10^5^ cells/mL in DMEM medium (Gibco). Next, cells were adhered to slide glass using Cytospin, and fixed in 4% formalin phosphate buffer. After proteolytic activation for 10 min by proteinase K (Dako), samples were treated with methanol containing 0.3% H_2_O_2_ for 20 min to remove endogenous peroxidase activity. PBS containing 5% skimmed milk was used to block nonspecific background staining. Polyclonal rabbit anti-human TTR antibody (Dako) was diluted (1:100) in Dako REAL Antibody Diluent (Dako), and used as the primary antibody. Envision System-HRP Labelled Polymer Anti-Rabbit (Dako) was used as the secondary antibody. The reaction was visualized using the Dako Envision + kit/HRP (DAB) (Dako). TTR^+^ cells in CD14^+^ monocytes, iPS-MLs and SH-SY5Y cells were counted. Briefly, five visual fields were randomly chosen from each stained section, and the TTR^+^ cell number in each visual field determined. The average number of TTR^+^ cells per visual field was calculated.

### MTS assay

After culturing iPS-MLs (1× 10^5^ cells/well) with the appropriate TTRs for 3 days, cell viability was measured by MTS assay. Briefly, a Cell Titer 96 Aqueous One Solution Cell Proliferation Assay kit (Promega, Madison, WI, USA) was used in each well of the cultured plate. After incubation for 1 h at 37°C, absorbance in each well was measured at 490 nm.

### Statistical analysis

For the ELISA assay, the Tukey post-hoc test after a separate two-way repeated-measures analysis of variance (ANOVA) was used. The pairwise *t*-test with Bonferroni correction after one-way ANOVA was used for the MTS assay. This analysis was performed using R version 3.2.1. For immunohistochemistry staining of heart tissue, repeated count immunostained cells were analyzed using the generalized Poisson mixed model. The proportion of repeated count TTR^+^ iPS-MLs, SH-SY5Y cells and CD14^+^ monocytes were analyzed using the mixed model. These analyses were performed using SAS Version 9.4. A value of *p* < 0.05 was considered statistically significant.

## Results

### Histopathological characteristics of FAP ATTR V30M patients

**Fig 1:**
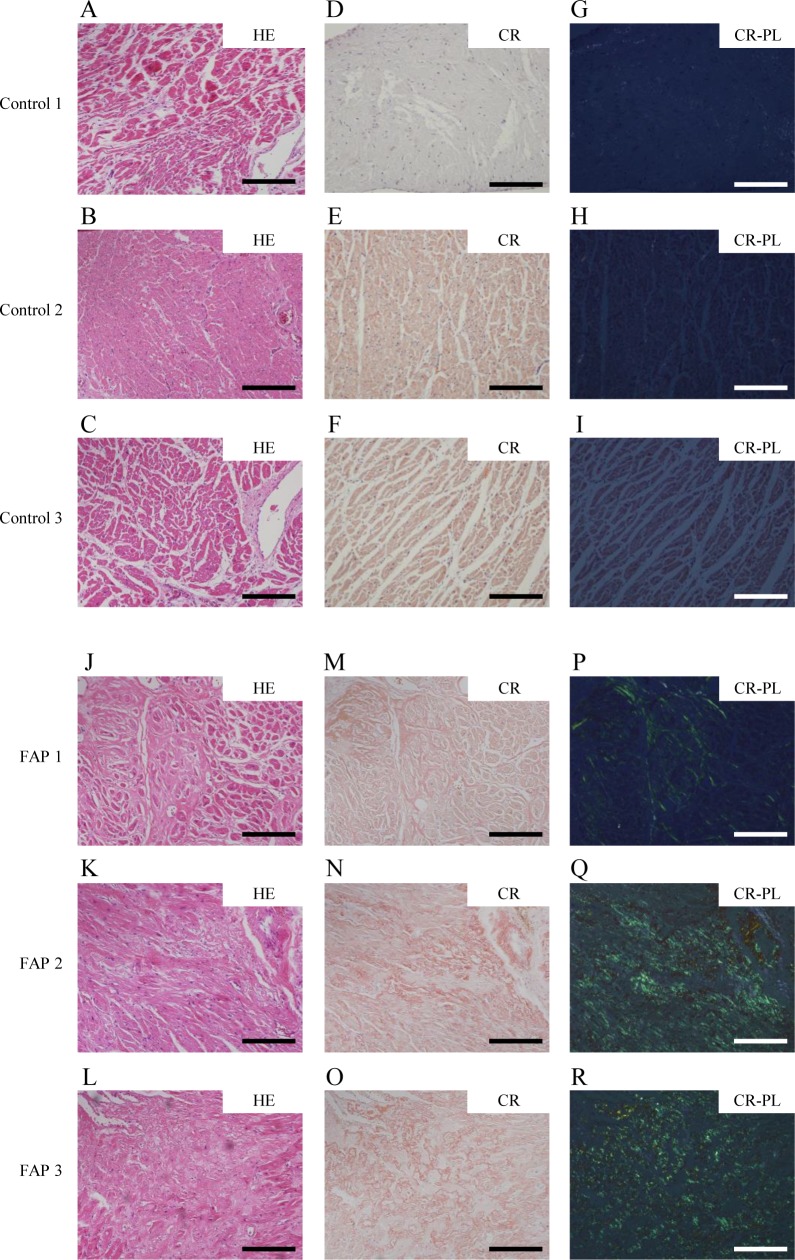
Histopathological characteristics in FAP ATTR V30M and control patients. Histopathological findings in heart tissue. HE-stained (A-C and J-L), Congo red-stained (D-F and M-O), and Congo red-stained tissue showing green birefringence under polarizing light (G-I and P-R) in control and FAP ATTR V30M patients. Control patient 1 (A, D, G), patient 2 (B, E, H), and patient 3 (C, F, I); and FAP patient 1 (J, M, P), patient 2 (K, N, Q), and patient 3 (L, O, R). Scale bars: 200 μm. Representative cases are shown.

The characteristics of FAP patients employed in this study are demonstrated in Table 1. To investigate the condition of macrophages in FAP, we analyzed the number of tissue-resident macrophages in the heart, which is one of the most TTR-derived amyloid fibril-laden organs. Moreover, inflammation causes recruitment of inflammatory cells, including macrophages, and affects the number and polarity of endogenous tissue-resident macrophages, although this process rarely occurs in the heart [35]. By performing HE and anti-CD3 staining, we first found that both control- and FAP-derived heart tissue do not contain migrating inflammatory cells such as T cells (Fig 1A–1C and 1J–1L, and S1 Fig). Next, heart tissue from control and FAP patients was stained with Congo red, as Congo red polarization confirms amyloid deposition. Although there was no amyloid deposition in control patients, mild or severe amyloid deposition was observed in heart tissue from all FAP patients (Fig 1D–1I and 1M–1R). Additionally, tissue destruction and myocardial cell death were observed, coincident with areas of severe amyloid deposition (data not shown).

### Decreased tissue-resident macrophages in heart tissue from FAP patients

**Fig 2:**
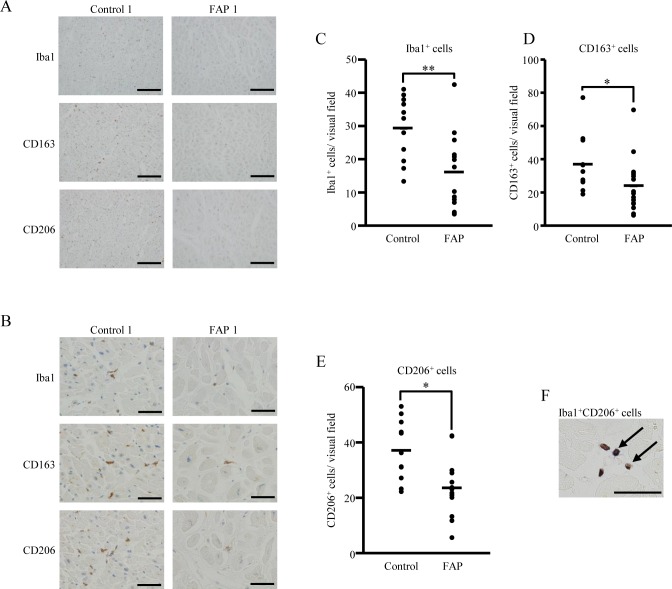
Lower number of tissue-resident macrophages in FAP ATTR V30M patients. (A, B) Heart tissue (FAP ATTR V30M patients, *n* = 16; control patients, *n* = 11) was stained with macrophage-related (Iba1) and inhibitory macrophage (CD163 and CD206) markers by immunohistochemistry. All slides shown are representative of each group. Lower (A) and higher (B) magnification views are shown. (C-E) Five visual fields in each stained section were randomly chosen, and the number of Iba1, CD163, and CD206-positive cells counted by two independent observers. Graphs show the average count number per visual field for each marker: Iba1 (C), CD163, (D) and CD206 (E). Repeated count immunostained cells were analyzed using the generalized Poisson mixed model, with *p < 0.05 and **p < 0.01 indicating significant differences. (F) Double immunohistochemical staining of Iba1 and CD206 in heart tissue of FAP ATTR V30M patients. Black arrows show double-immunostained cells. Bars indicate 200 μm (A) and 50 μm in (B, F).

To detect heart-tissue resident macrophages, we performed immunohistochemistry using Iba and CD68 antibodies, which are both well-known macrophage markers [36, 37]. The number of Iba1- and CD68-positive macrophages in FAP-derived heart tissue was decreased compared with control patients (Fig 2A–2C and S2 Fig). Tissue-resident macrophages are reported to be inhibitory macrophages [24]. Therefore, we investigated the phenotype of tissue-resident macrophages in control and FAP patients. To identify these macrophages, we immunohistochemically stained heart tissue using the inhibitory macrophages markers, CD163 and CD206 (Fig 2A and 2B). The number of both CD163- and CD206-positive cells was decreased in FAP patients compared with controls (Fig 2D and 2E). Additionally, we confirmed that all Iba1-positive cells were identical to CD163- or CD206-positive cells by double immunohistochemical staining (Fig 2F and S2 Fig). Taken together, these results suggest that tissue-resident macrophages are decreased in FAP patients.

### Reduced intracellular TTR in CD14^+^ monocytes from FAP patients

**Fig 3:**
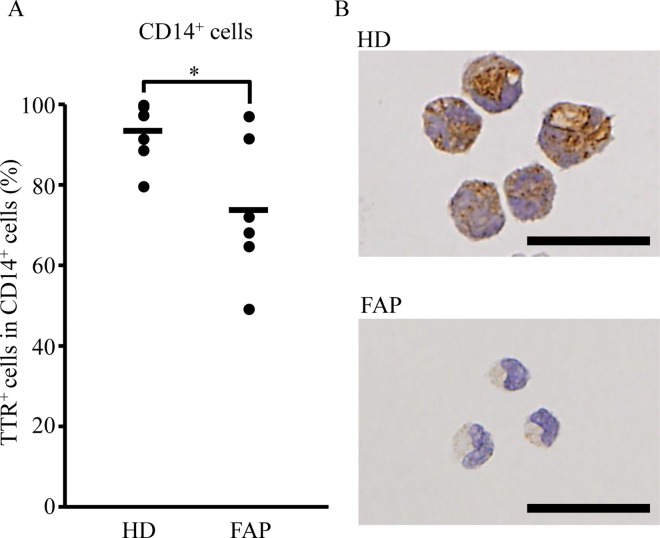
Decreased frequency of intracellular TTR in CD14^+^ monocytes from FAP ATTR V30M patients. CD14^**+**^ monocytes were isolated from PBMC of HD (*n* = 7) or FAP ATTR V30M patients (*n* = 6) using Ficoll-Paque and magnet bead-conjugated anti-human CD14 antibodies. (A, B) Separated CD14^**+**^ monocytes were stained with an anti-human TTR antibody. Five visual fields in each stained section were randomly chosen, and the number of TTR-positive CD14^**+**^ monocytes counted by three independent observers. The average count number per visual field is shown. The bar graph (A) shows the frequency of TTR^+^ cells in CD14^+^ monocytes. Photographs (B) show representative data for each group. Scale bars indicate 25 μm. (A, B) The proportion of repeated count TTR^+^ CD14^+^ monocytes were analyzed using the mixed model, with *p < 0.05 indicating a significant difference.

Monocytes are reported to partly differentiate into tissue-resident macrophages [38], therefore, we focused our attention on blood monocytes. CD14^+^ monocytes are well known to subdivide into CD14^high^CD16^-^, CD14^high^CD16^+^, and CD14^low^CD16^+^ monocyte subsets [39]. Thus, we investigated the proportion of these three subsets between HD and FAP patients. There were no significant differences in the proportion of these three subsets or CD163 expression in each subset between both groups (S3 Fig). Next, isolated CD14^+^ monocytes from HD and FAP patients were applied to cytospin preparations and stained with an anti-TTR antibody. Consequently, CD14^+^ monocytes from FAP patients were shown to have lower intracellular TTR immunoreactivity compared with HD (Fig 3A and 3B). Overall, these results suggest that CD14^+^ monocytes may affect TTR clearance, with a decreased ability in FAP patients.

### Expression of CD163 and CD206 on iPS-MLs

We observed a lower number of inhibitory macrophages in heart tissue from FAP patients compared with control patients. Further, inhibitory macrophages are reported to have anti-inflammatory effects and induce tissue repair [24]. These results suggest that decreased tissue-resident inhibitory macrophages in FAP patients may exacerbate TTR-derived amyloid deposition. Consequently, it is possible that inhibitory macrophage transfer may be useful as a therapeutic approach for FAP. In this regards, we have previously generated iPS-MLs that function as macrophage-like myeloid lineage cells, and shown that they exhibit efficient β-amyloid phagocytic activity in a mouse model of Alzheimer’s disease [32]. Thus here, we determined if iPS-MLs also have phagocytic capacity for TTR-derived amyloid fibrils.

**Fig 4:**
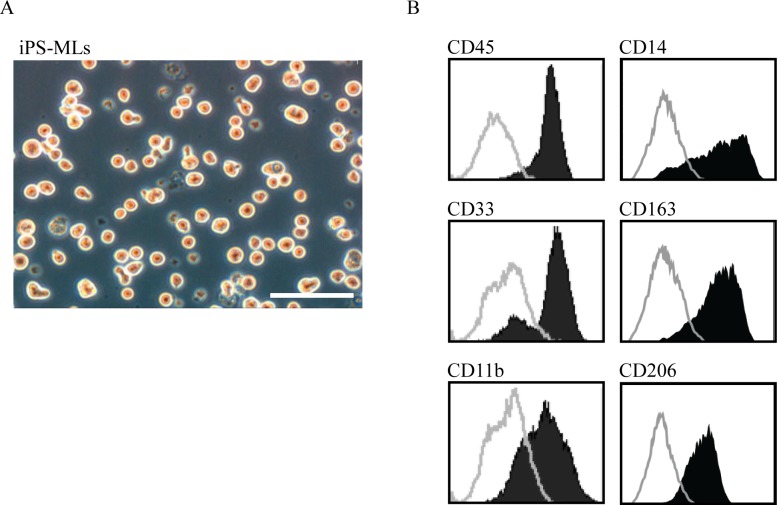
Morphology and expression of inhibitory macrophage markers in iPS-MLs. (A) iPS-ML morphology is shown. (B) Flow cytometry analysis shows expression of myeloid markers including CD45, CD33, CD14, and CD11b on iPS-MLs. Inhibitory macrophage markers, CD163 and CD206, were identified on iPS-MLs. Staining profiles of specific (black areas) and isotype-matched control (gray lines) monoclonal antibodies are shown. Data are representative of three independent experiments. Scale bars: 100 μm.

First, we examined the phenotype of iPS-MLs by flow cytometry. After confirming cell morphology and hematopoietic and myeloid markers, such as CD45, CD33, CD14, and CD11b (Fig 4A and 4B), we detected expression of CD163 and CD206 on iPS-MLs (Fig 4B). Our results suggest that iPS-MLs belong to the inhibitory macrophage class.

### *In vitro* degradation of native and aggregated TTR by iPS-MLs

**Fig 5:**
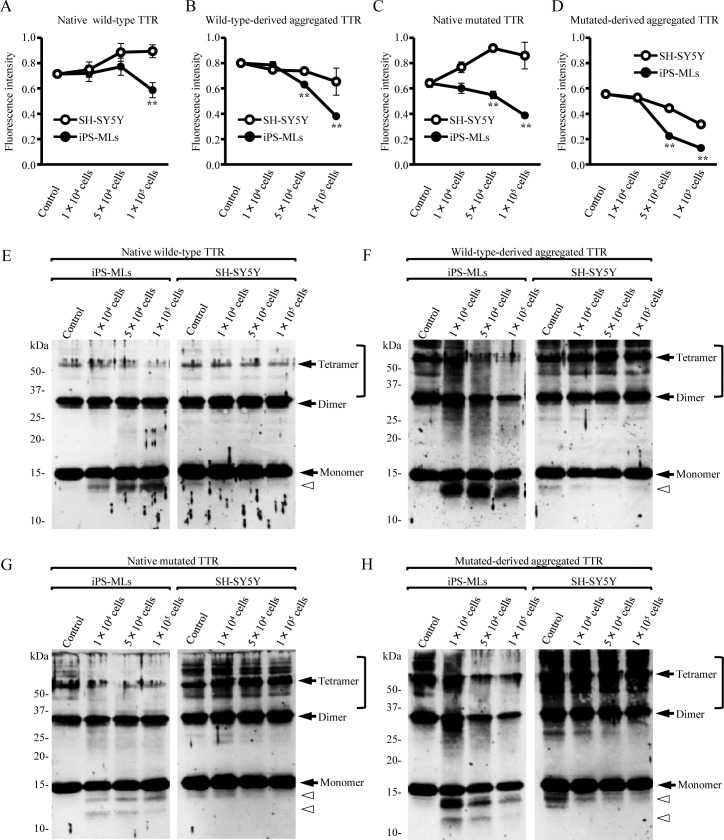
iPS-MLs degrade aggregated TTR. (A-H) iPS-MLs or control SH-SY5Y cells (1 × 10^4^, 5 × 10^4^, or 1× 10^5^ cells/well) were cultured with native wild-type, mutated TTR, wild-type-derived, and mutated-derived aggregated TTR for 3 days. Detection of TTR in each culture supernatant was determined by ELISA (A-D) or western blot analysis (E-H). (A-B and E-F) Native wild-type and wild-type-derived aggregated TTR. (C-D and G-H) Native mutated and mutated-derived aggregated TTR. (A-D) Tukey post-hoc test after separate two-way repeated-measures analysis of variance. **p < 0.01. Data are representative of two independent experiments. (E-H) Black arrows and white arrowheads indicate full-length TTR and truncated TTR, respectively. Square brackets indicate polymerized TTR. Mutated TTR represents V30M TTR.

We next determined if iPS-MLs reduce TTR *in vitro*. To exclude the influence of serum, which naturally contains wild-type TTR, iPS-MLs or SH-SY5Y cells (as controls) were cultured in the presence of native wild-type or mutated TTR under serum-free conditions. After 3 days, TTR in the culture supernatant was quantified by ELISA. Concentration of both native wild-type and mutated TTR decreased in a cell number-dependent manner in iPS-MLs compared with SH-SY5Y cells (Fig 5A and 5C). This tendency was similar in iPS-ML culture in the presence of both wild-type- and mutated-derived aggregated TTR (Fig 5B and 5D).

Additionally, we confirmed degraded TTR in culture supernatants by western blotting. Truncated TTR was detected in iPS-ML culture conditions with all types of TTR (Fig 5E–5H), and possibly reflects TTR degradation owing to iPS-MLs. Under native wild-type and mutated TTR culture conditions, neither iPS-MLs nor SH-SY5Y cells decreased full-length TTR in a cell number-dependent manner (Fig 5E and 5G). In contrast, compared with native TTR culture conditions, wild-type- and mutated-derived aggregated TTR culture conditions resulted in the appearance of polymerized TTR fibrils. Furthermore, iPS-MLs significantly degraded polymerized TTR fibrils and truncated TTR regardless of TTR type. These phenomena were dependent on iPS-ML number (Fig 5F and 5H). These results suggest that iPS-MLs degrade both wild-type and mutated TTR, with a greater degradation capability for polymerized TTR fibrils.

### Proliferated cell viability of iPS-MLs in the presence of wild-type TTR

Next, to determine the toxicity of TTR on iPS-MLs, we performed the MTS assay. Viability of iPS-MLs cultured in the presence of native or aggregated wild-type TTR increased compared with those cultured without TTR (S4 Fig).In contrast, mutated and mutated–derived aggregated TTR did not influence iPS-MLs (S4 Fig).

### Intracellular uptake of TTR in iPS-MLs

**Fig 6:**
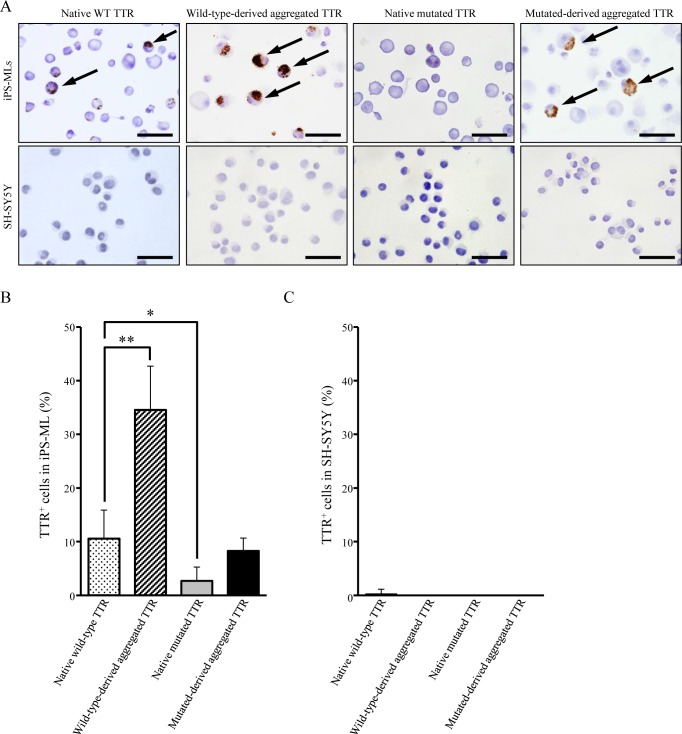
Intracellular TTR immunoreactivity in iPS-MLs. iPS-MLs or control SH-SY5Y cells (1 × 10^4^, 5 × 10^4^, or 1× 10^5^ cells/well) were cultured with native wild-type, wild-type-derived aggregated, native mutated, and mutated-derived aggregated TTR as described in the legend for Fig 5. Cultured iPS-MLs and SH-SY5Y cells were stained with TTR antibody by immunohistochemistry. (A) All slides shown are representative for each group. Arrows indicate intracellular TTR. Scale bars: 50 μm. (B-C) Five visual fields in each stained section were randomly chosen, and the number of TTR^+^ cells counted by two observers. Bar graphs show the frequency of TTR^+^ cells in (B) iPS-MLs and (C) SH-SY5Y cells. The proportion of repeated count TTR^+^ cells were analyzed using the mixed model, with *p < 0.05 and **p < 0.01 indicating a significant difference.

To determine if iPS-MLs phagocytize aggregated TTR, iPS-MLs and SH-SY5Y cells cultured with native or aggregated TTR were stained with an anti-TTR antibody. Although native wild-type and mutated-derived aggregated TTR was slightly internalized by iPS-MLs, intake of mutated TTR was (Fig 6A and 6B). In addition, intracellular TTR was predominantly identified in iPS-MLs cultured with aggregated wild-type TTR compared with other TTR-culture conditions. Conversely, SH-SY5Y cells showed no intracellular TTR immunoreactivity under any culture conditions (Fig 6A and 6C). Taken together, these results indicate that iPS-MLs mainly endocytose wild-type-derived aggregated TTR.

## Discussion

To examine tissue-resident macrophages in FAP patients, we chose heart tissue as an organ of TTR deposition for the following two reasons. The first being that heart tissue is one of the most TTR-derived amyloid fibril-laden organs [2]. Indeed, cardiac amyloidosis in FAP patients progresses regardless of LT [21, 22]. Additionally, senile systemic amyloidosis, which is a non-hereditary disease, causes TTR-derived cardiac amyloidosis by idiopathic deposition of wild-type TTR [40]. In contrast, no or only mild TTR-derived amyloid deposits are found in the perivascular lesions of liver, spleen, and lymph nodes in FAP patients [41, 42]. In particular, although hepatocytes produce the majority of systemic TTR, they are not usually affected by this [43]. In this regards, we focused on the heart, with fewer TTR-derived amyloid fibrils in FAP patients and relatively abundant tissue-resident macrophages. Kupffer cells are present among hepatocytes, and spleen or lymph nodes also store macrophages [44, 45]. To determine if macrophages are involved in clearance of TTR and aggregated TTR, Misumi *et al*. recently reported that macrophages and fibroblasts play a central role in endocytosis and degradation of aggregated TTR in FAP patients [46]. Accordingly, we investigated whether or not tissue-resident macrophages are decreased in heart tissue, which has relatively fewer macrophages and higher TTR deposition.

Our second reason was that heart tissue inflammation (e.g., myocarditis) is rare. Under inflammatory conditions, inflammatory cells (including macrophages) are easily recruited into the inflammatory site, and macrophages shift in polarity to inflammatory macrophages, and thereby release inflammatory cytokines [38, 47]. In contrast, under recovery conditions, inhibitory macrophages are dominant and have anti-inflammatory effects [24, 47]. Furthermore, inhibitory macrophages are involved in the removal of debris, angiogenesis, and tissue remodeling [24]. Because we aimed to identify tissue-resident macrophages in as physiological a condition as possible, we chose heart tissue, which is comparatively insulated from the influence of inflammation.

Based on these findings, we hypothesized that FAP patients would display quantitative or qualitative abnormalities in tissue-resident macrophages, especially inhibitory macrophages, and these abnormalities may accelerate TTR-derived amyloid deposition in some organs.

In this present study, our analysis of heart tissue showed a significantly lower number of tissue-resident macrophages in non-LT FAP patients compared with control patients. This suggests that quantitative reduction of tissue-resident macrophages partly triggers TTR deposition in heart tissue, which originally had fewer macrophages compared with the liver, spleen, and lymph nodes.

Tissue-resident macrophages originate from embryonic precursors derived from the yolk sac and can self-replenish. These macrophages are known as the inhibitory phenotype [23]. In contrast, macrophages can be partly developed from circulating blood monocytes [26, 38], and monocyte-derived macrophages include both inflammatory and inhibitory phenotypes [26, 38]. We found that tissue-resident macrophages in heart tissue from FAP patients were of the inhibitory phenotype. Therefore, the inhibitory macrophages we detected are more likely derived from the yolk sac rather than differentiated monocytes. In addition, qualitative abnormalities in decreased inhibitory macrophages may result in inadequate care for this regional organ, and consequently, TTR deposition may be accelerated. However, the cause of the macrophages decrease in heart tissue from FAP patients is not obvious. Further research and approaches are needed.

As described above, tissue-resident macrophages, at least in part, differentiate from monocytes [26, 38]. CD14^+^ monocytes can be divided into three distinct subsets: CD14^high^ CD16^-^, CD14^high^CD16^+^, and CD14^low^ CD16^+^ monocytes, which have different capacities for modulating inflammatory responses [48, 49]. Accordingly, we next determined if there are differences in the frequency of each monocyte subset between HD and FAP patients, but found they were similar between both groups. In addition, CD163 was also equally expressed on each subset. In contrast, the intracellular TTR proportion in CD14^+^ monocytes from FAP patients was decreased compared with HD. These results suggest that FAP patients have insufficient TTR processing ability in the periphery. However, there was no difference in CD163 expression between HD and FAP patients. Moreover, because CD163 is a hemoglobin scavenger receptor [50], it is not directly involved in TTR uptake. It was recently reported that administration of the interleukin-1 antagonist, anakinra, induced inhibition of TTR deposition at the sciatic nerve in a FAP model mouse [51]. Moreover, interleukin-6 expression is highly expressed in chondrocytes in the presence of mutated V122I TTR [52]. These results suggest the possibility that mutated TTR produces pro-inflammatory cytokines, which may affect both tissue-resident macrophages and peripheral monocytes. Further studies are needed to clarify the mechanism of decreased inhibitory tissue-resident macrophages, and reduced endocytosis of intracellular TTR in peripheral monocytes of FAP patients.

We have recently shown that iPS-MLs are a potential therapeutic agent in the treatment of Alzheimer’s disease [31, 32], which causes accumulation of misfolded β-amyloid. FAP is also a disorder characterized by deposition of misfolded TTR, therefore we investigated the possibility that iPS-MLs are a useful tool for FAP. We found that iPS-MLs are inhibitory macrophages and degrade aggregated, especially polymerized TTR, in a cell number-dependent manner. Furthermore, intracellular TTR was detected in iPS-MLs cultured with wild-type-derived TTR compared with mutated-derived TTR, and iPS-MLs exhibit more phagocytic ability for aggregated TTR. In addition, iPS-MLs cultured with wild-type-derived TTR showed increased cell viability. In contrast, the propensity of iPS-MLs for mutated-derived TTR was not as great, and endocytosis of this TTR was poor. Further, mutated-derived TTR did not affect cell viability of iPS-MLs. These results suggest that iPS-MLs, which show improved cell viability with wild-type TTR, are able to more readily endocytose this type and do not function well with mutated TTR. However, truncated TTR appeared in iPS-MLs cultured with all types of TTR. These results indicate that iPS-MLs degrade both native and aggregated TTR.

Taken together, we have demonstrated the intrinsic function of iPS-MLs to be the inhibitory phenotype, leading to degradation and incorporation of TTR *in vitro*. However, we have not yet shown if iPS-MLs actually reduce TTR deposition *in vivo*. Therefore, although further investigations are required, considering the recent advances in human iPS cell-related technologies, pluripotent stem cell-derived myeloid lineages may be a promising means for the treatment of FAP.

## Supporting Information

S1 Fig Staining of T cells in FAP ATTR V30M and control patients. Heart tissue (FAP ATTR V30M patients, *n* = 16; control patients, *n* = 11) was stained with a T-cell marker (CD3) by immunohistochemistry. Representative cases of control (A-C) and FAP patients (D-F) are shown. Bars indicate 200 μm.
(TIF)

S2 Fig Decreased number of tissue-resident M2 macrophages in FAP ATTR V30M patients. Heart tissue from FAP ATTR V30M (*n* = 16) and control (*n* = 11) patients was used (as described in the legend for Fig 2). (A, B) Representative slides for each group are shown, with lower (A) and higher (B) magnification views. (C) Average count number of CD68-positive cells per visual field. Repeated count CD68-positive cells were analyzed using the generalized Poisson mixed model, with **p < 0.01 indicating a significant difference. (D) Double immunohistochemical staining of Iba1 and CD163 in heart tissue from FAP ATTR V30M patients. Black arrows show double-immunostained cells. Bars indicate 200 μm (A) and 50 μm in (B, D).
(TIF)

S3 Fig CD163 expression on CD14^high^CD16^-^, CD14^high^CD16^+^, and CD14^low^CD16^+^ monocytes from HD and FAP patients. PBMC were collected from HD (*n* = 15) or FAP patients (*n* = 15: 13 FAP ATTR V30M, one Y114C, and one I107V). (A) The proportion of CD14^high^ CD16^-^, CD14^high^CD16^+^, and CD14^low^ CD16^+^ monocytes in total CD14^**+**^ monocytes was determined by flow cytometry. (B) Similarly, CD163 expression in the three monocyte subsets was determined by flow cytometry.
(TIF)

S4 Fig Cytotoxicity evaluation of TTR in iPS-MLs. iPS-MLs (1× 10^5^ cells/well) were cultured with native wild-type, mutated, wild-type-derived, and mutated-derived aggregated TTR. After 3 days, the viability of each group was evaluated by the MTS assay. Data were analyzed using the pairwise *t*-test with Bonferroni correction after one-way ANOVA, with **p < 0.01 indicating a significant difference. Data are representative of four independent experiments.
(TIF)
