# Supplementary figures and images for: Involvement of Macrophages in the Pathogenesis of Familial Amyloid Polyneuropathy and Efficacy of Human iPS Cell-Derived Macrophages in Its Treatment
Source: PLoS One. 2016 Oct 3;11(10):e0163944. doi: 10.1371/journal.pone.0163944 (PMC5047455; doi:10.1371/journal.pone.0163944)

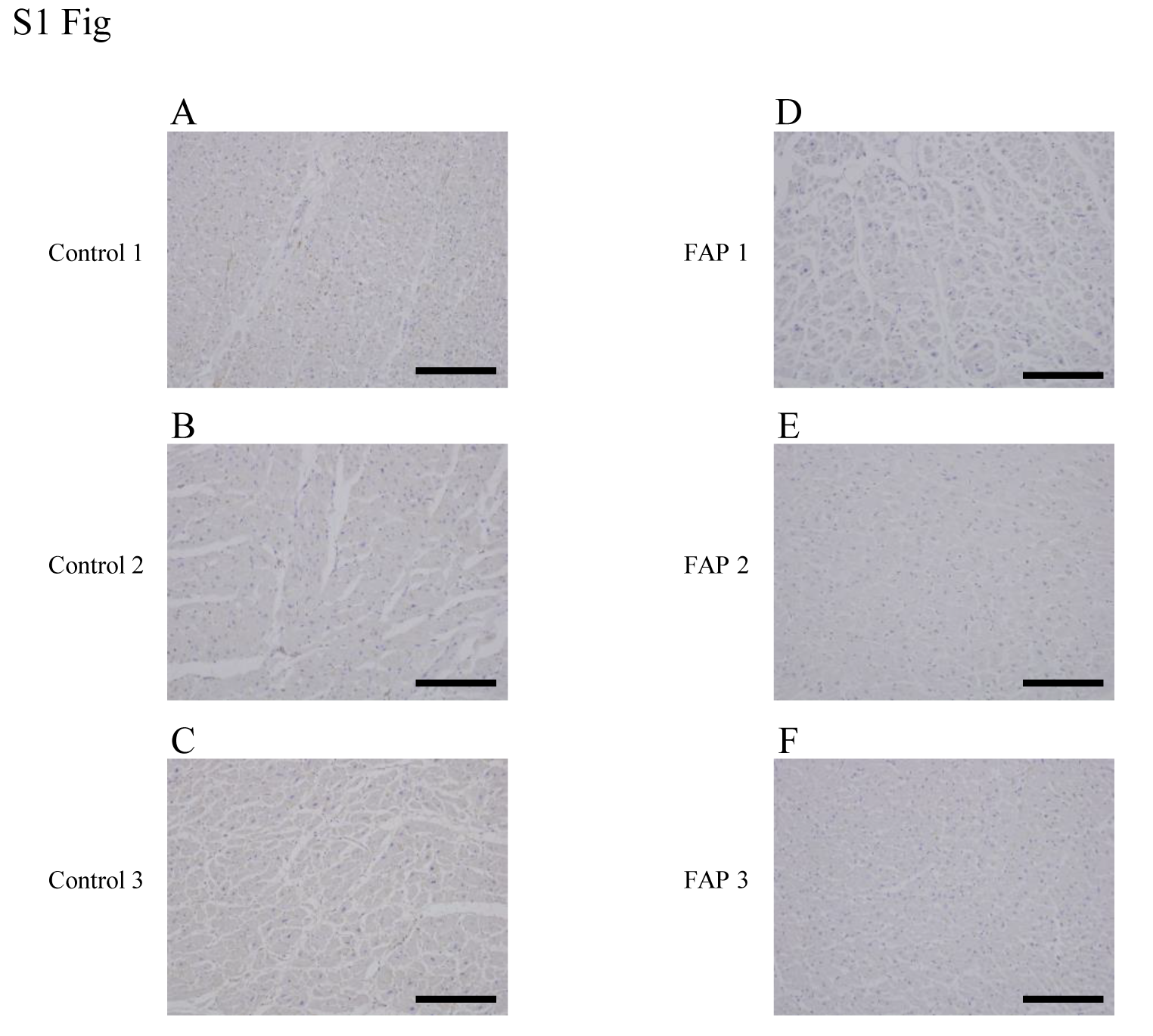

Supplement: S1 Fig — Heart tissue (FAP ATTR V30M patients, n = 16; control patients, n = 11) was stained with a T-cell marker (CD3) by immunohistochemistry. Representative cases of control (A-C) and FAP patients (D-F) are shown. Bars indicate 200 μm. (TIF) [file pone.0163944.s001.tif]

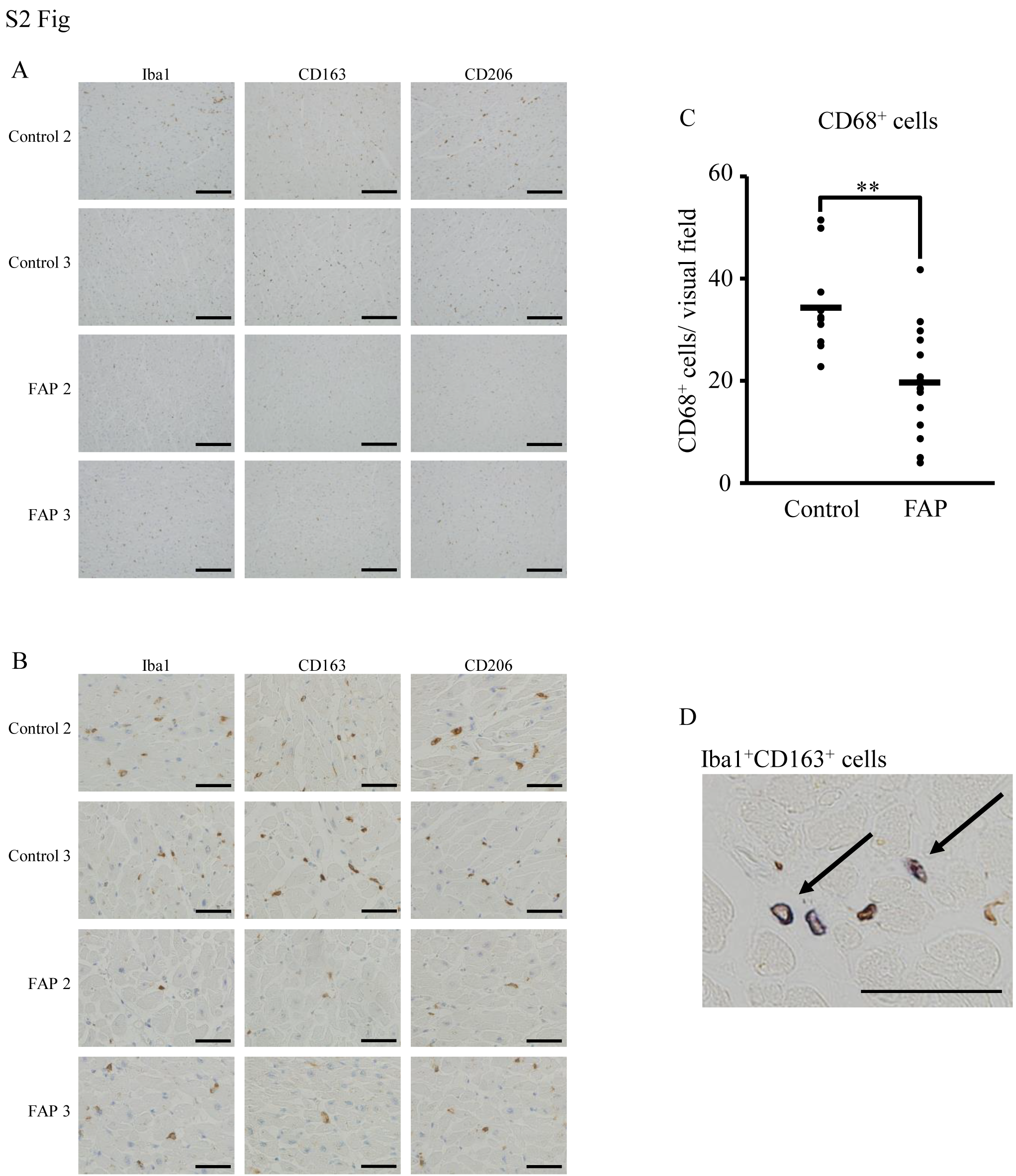

Supplement: S2 Fig — Heart tissue from FAP ATTR V30M (n = 16) and control (n = 11) patients was used (as described in the legend for Fig 2). (A, B) Representative slides for each group are shown, with lower (A) and higher (B) magnification views. (C) Average count number of CD68-positive cells per visual field. Repeated count CD68-positive cells were analyzed using the generalized Poisson mixed model, with **p < 0.01 indicating a significant difference. (D) Double immunohistochemical staining of Iba1 and CD163 in heart tissue from FAP ATTR V30M patients. Black arrows show double-immunostained cells. Bars indicate 200 μm (A) and 50 μm in (B, D). (TIF) [file pone.0163944.s002.tif]

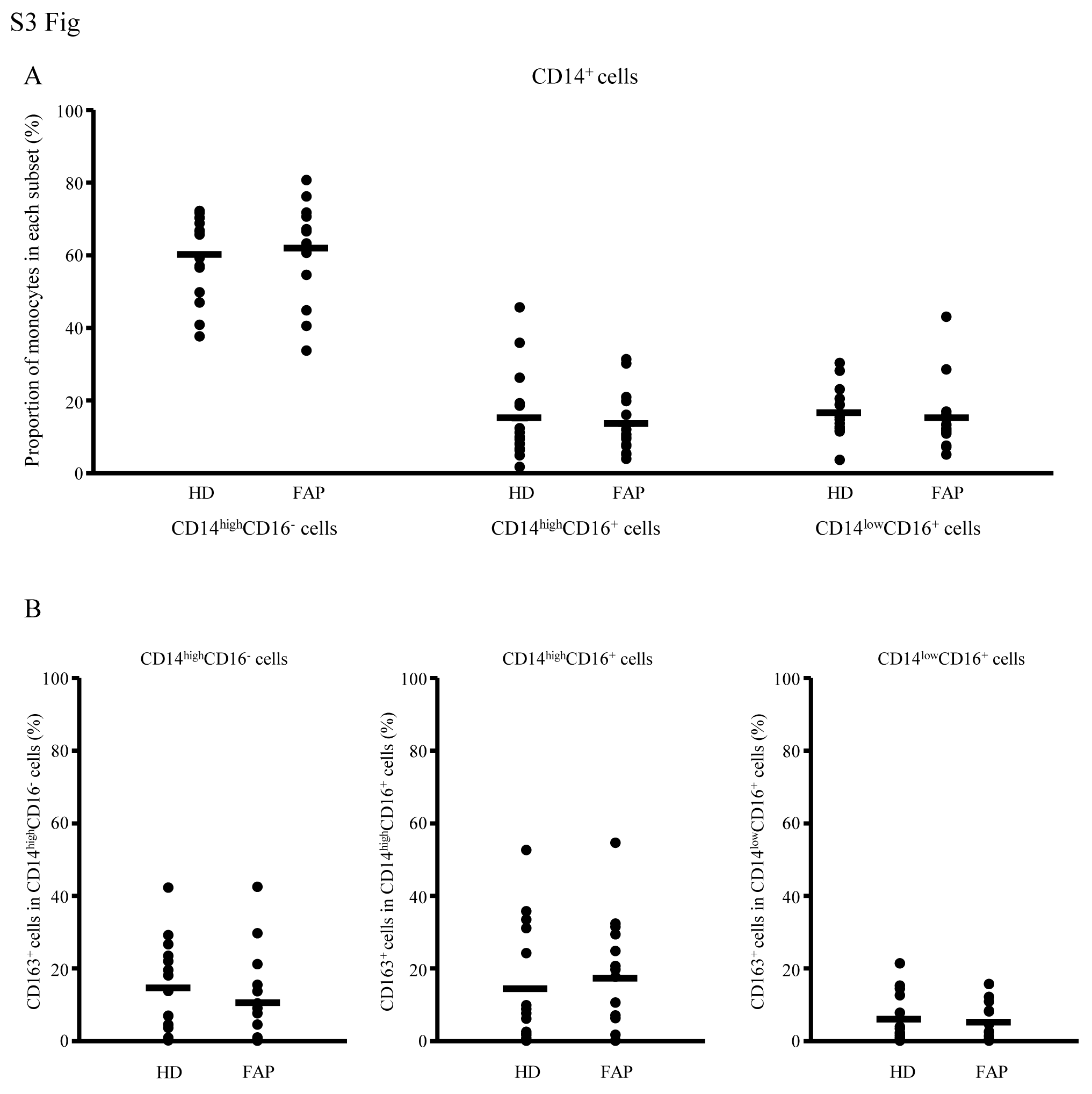

Supplement: S3 Fig — PBMC were collected from HD (n = 15) or FAP patients (n = 15: 13 FAP ATTR V30M, one Y114C, and one I107V). (A) The proportion of CD14high CD16-, CD14highCD16+, and CD14low CD16+ monocytes in total CD14+ monocytes was determined by flow cytometry. (B) Similarly, CD163 expression in the three monocyte subsets was determined by flow cytometry. (TIF) [file pone.0163944.s003.tif]

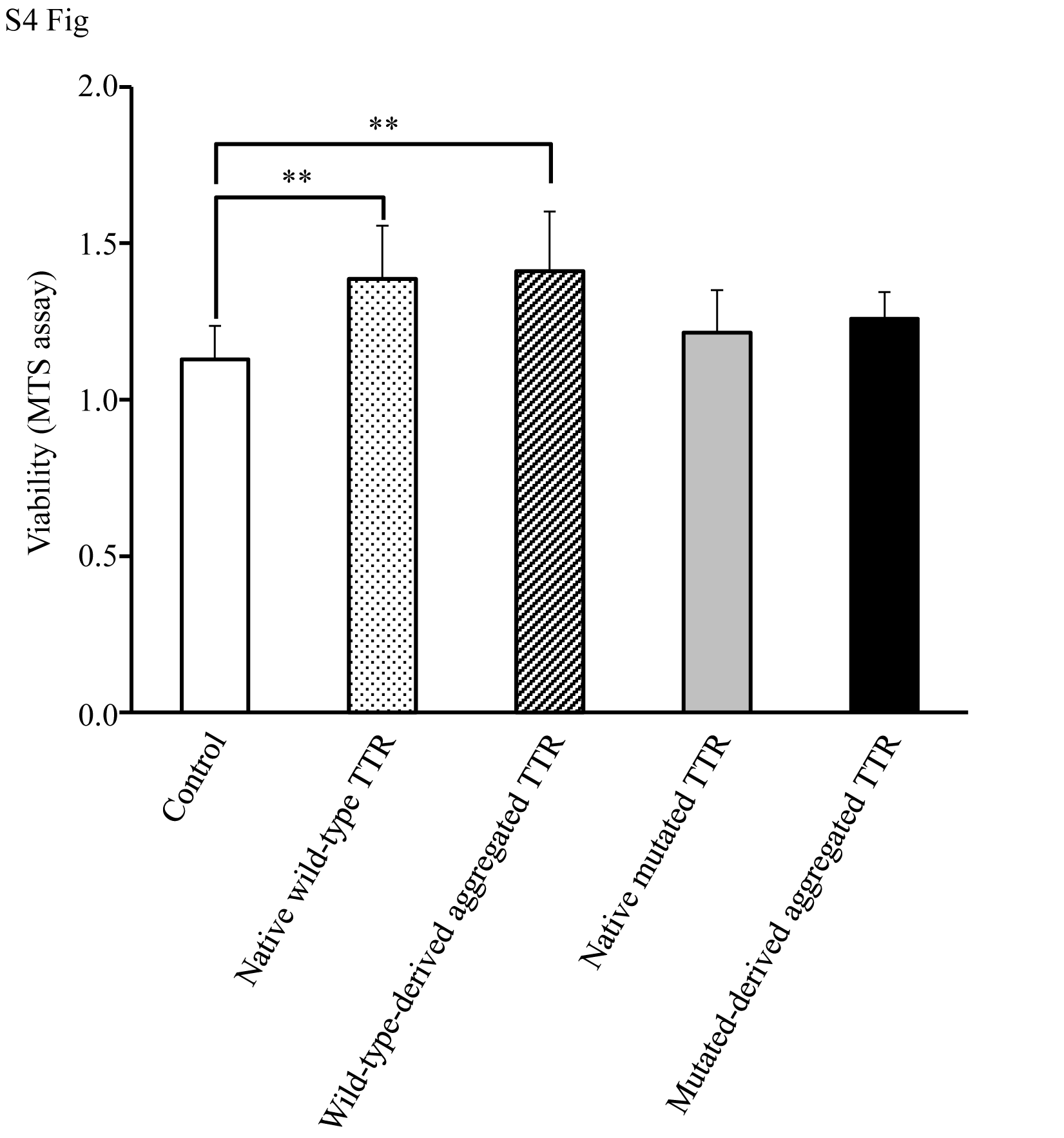

Supplement: S4 Fig — iPS-MLs (1× 10^5^ cells/well) were cultured with native wild-type, mutated, wild-type-derived, and mutated-derived aggregated TTR. After 3 days, the viability of each group was evaluated by the MTS assay. Data were analyzed using the pairwise t-test with Bonferroni correction after one-way ANOVA, with **p < 0.01 indicating a significant difference. Data are representative of four independent experiments. (TIF) [file pone.0163944.s004.tif]
